# A Comprehensive Workflow of Mass Spectrometry-Based Untargeted Metabolomics in Cancer Metabolic Biomarker Discovery Using Human Plasma and Urine

**DOI:** 10.3390/metabo3030787

**Published:** 2013-09-11

**Authors:** Wei Zou, Jianwen She, Vladimir V. Tolstikov

**Affiliations:** 1 California Department of Public Health, 850 Marina Bay Parkway, G365, Richmond, CA 94804, USA; E-Mail: Jianwen.she@cdph.ca.gov; 2 Tailored Therapeutics, Eli Lilly and Company, Lilly Corporate Center, DC 0714, Indianapolis, IN 46285, USA

**Keywords:** untargeted metabolomics, pathway analysis, HILIC, RP, TOF, FT-ICR, Orbitrap, QTrap, mass spectrometer, cancer, human, plasma, urine

## Abstract

Current available biomarkers lack sensitivity and/or specificity for early detection of cancer. To address this challenge, a robust and complete workflow for metabolic profiling and data mining is described in details. Three independent and complementary analytical techniques for metabolic profiling are applied: hydrophilic interaction liquid chromatography (HILIC–LC), reversed-phase liquid chromatography (RP–LC), and gas chromatography (GC). All three techniques are coupled to a mass spectrometer (MS) in the full scan acquisition mode, and both unsupervised and supervised methods are used for data mining. The univariate and multivariate feature selection are used to determine subsets of potentially discriminative predictors. These predictors are further identified by obtaining accurate masses and isotopic ratios using selected ion monitoring (SIM) and data-dependent MS/MS and/or accurate mass MS^n^ ion tree scans utilizing high resolution MS. A list combining all of the identified potential biomarkers generated from different platforms and algorithms is used for pathway analysis. Such a workflow combining comprehensive metabolic profiling and advanced data mining techniques may provide a powerful approach for metabolic pathway analysis and biomarker discovery in cancer research. Two case studies with previous published data are adapted and included in the context to elucidate the application of the workflow.

## 1. Overview

Over the last several decades there has been significant progress in understanding cancer pathology, but cancer is still a devastating disease with high morbidity and mortality if diagnosed too late. The general consensus is that early detection is one of the key aspects to prevent or slow down the development of cancer in patients. However, currently available biomarkers lack sensitivity and/or specificity for early detection of cancer. In the near future, the notions of system biology and personalized medicine are expected to fundamentally change our views on health and diseases; including cancer [1,2,3]. The term “system biology” has been coined to integrate data generated by all the “omics” platforms, thus taking advantage of the fast paced IT industry.

Currently there are challenges in each realm of “omics”. In metabolomics studies, samples are highly complex, biologically variant, and with a large dynamic range of concentrations of active components. Such factors as well as high degree of structural diversity of metabolites present challenges to separation, detection, and data analysis. Multiple techniques have been be used in metabolomics, such as nuclear magnetic resonance spectroscopy (NMR), gas chromatography-mass spectrometry (GC-MS), liquid chromatography-mass spectrometry (LC-MS), and capillary electrophoresis-mass spectrometry (CE-MS), each with its own advantages and drawbacks. In order to identify various nontrivial metabolites with different polarity and molecular weight, all of the above-mentioned techniques are needed. Therefore, both LC-MS and GC-MS platforms are used in our untargeted metabolomics studies. The general steps include sample preparation, instrumental analysis, data mining, annotation and identification of feature components, secondary metabolite profiling using predictive multiple reaction monitoring (pMRM), and metabolic pathway analysis.

### 1.1. Hyphenated Separation and Detection Techniques

NMR is a non-destructive analytical technique which is good at detecting positional isomers and has been used to analyze flux rates of metabolic pathways. However, it is difficult to analyze traditional NMR spectra of complex mixtures because of its relatively low sensitivity at the micro-molar range; leaving low-abundant metabolites undetected. Comparatively, MS provides better sensitivity, a wider range of covered metabolites, and direct-injection can analyze a large number of metabolites in a very short amount of time. Direct injection MS can be hassled with ion suppression caused by complex sample matrices, where the signal of many analytes with low ionization efficiencies cannot be detected. To avoid these problems, MS is often hyphenated to GC or LC to decrease sample complexity.

GC-MS is a sensitive and robust separation technique with established applications in the field of metabolomics. GC-MS has good separation resolution due to the long capillary column and readily available commercial mass spectra databases, thus annotation of unknown peaks is quick [4]. GC-MS typically uses electron impact ionization (EI) which is less prone to sample matrix effects, ionizes most compounds with relatively high efficiency, and generates instrument-independent (within the same type of mass spectrometer) mass spectra for library build-up. Mass spectra acquired from a single quadruple MS are typically different from those acquired from an ion trap MS; therefore, libraries built on different types of MS may not be generalized. GC-MS can detect metabolites such as amino acids, organic acids, carbohydrates, phosphorylated metabolites, fatty acids, and cholesterol. However, GC-MS is not suitable for analysis of large or thermo labile compounds such as nonpolar intact lipids, nucleotides, nucleotide diphosphates, cofactors, or oligosaccharides.

Contrary to GC-MS, LC-MS usually does not require derivatization, has different types of columns for separation, and comes with large sample loading capacity. Reverse-phase chromatography (RP) is a mature technique for separation of non-polar compounds. Monolithic capillary columns [5,6,7,8,9,10,11] and ultra-performance liquid chromatography (UPLC) [12] have introduced high chromatographic peak resolution to LC, which in the past could be reached only by capillary GC columns. With a high resolution LC coupled to a high resolution MS of a wide mass scan range, high molecular weight of nonpolar compounds can be detected by RP-LC-MS, such as glycerolipids, phospholipids, fatty acids, bile acids, and sterols. However, RP chromatography is not easily applicable to separate highly polar compounds. Hydrophilic interaction chromatography (HILIC) is recommended to separate simple and complex carbohydrates, amino acids, glycosides, and other natural polar products [13,14,15,16,17,18,19,20]. We found that HILIC LC-ESI-MS technique is more informative than other technology platforms in analyzing human urine and plasma samples. This is reasonable because most compounds in human urine or plasma are water-soluble, therefore, are more suitable for HILIC-LC separation rather than for RP-LC separation.

Coupling the above-mentioned separation techniques to MS provides extremely versatile and valuable tool for metabolomics studies [10,11,15,21,22]. LC-MS typically uses electrospray ionization (ESI) that is prone to ion suppression/enhancement; therefore, good chromatographic separation is essential. Atmospheric-pressure chemical ionization (APCI) and atmospheric-pressure photoionization (APPI) have less sample matrix effects compared to ESI and are good for thermally-stable non-polar compounds; for that reason, both APCI and APPI have been used widely in lipidomics.

Ion chromatography (IC) is regularly used to separate organic anions and carbohydrates, but coupling to MS is not direct due to high concentrations of non-volatile inorganic salts (like NaOH or KOH) in mobile phases. However, with current commercially available post-column ion suppressors (such as Dionex ASRS 300) installed on an IC, inorganic salt ions can be exchanged out of the eluent so that MS detection will not be disturbed. For a typical IC-ESI-MS setup, it is necessary to have a post-column pump infusing acetonitrile or methanol at a certain flow rate to maintain ionization efficiency. Although complex instrumentation is necessary, IC-ESI-MS requires less column equilibration time and generates better peak shapes compared to HILIC-ESI-MS. In addition, IC can be directly coupled with inductively-coupled plasma (ICP)-MS without a post-column pump, providing sensitive information of elemental speciation complementary to information of molecular speciation generated from ESI-MS. We believe that the future optimal instrumental setup of untargeted metabolomics and proteomics in blood plasma and urine will be a capillary IC separating polar compounds coupled with multi-dimensional detectors in parallel using a splitter: an ESI-MS for molecular species screening, an ICP-MS for elemental species scanning, and a coulometric electrochemical array detector for isomer elucidation.

### 1.2. Untargeted Metabolic Profiling

Sample preparation is very important to the success of metabolomic analysis. Live tissues, organs or fluid should be deep flash frozen prior to extraction to prevent enzymatic alteration of components. It is recommended that multiple aliquots are stored at –80 °C, each time one aliquot is thawed overnight at 4 °C in the refrigerator, and multiple freeze/thaw cycles should be avoided. Samples must always be kept on ice when they are not in the refrigerator. To prevent bacterial growth in samples during storage, sodium azide may be added. The results from naturally clotting serum should not be mixed with those from plasma. Although lithium heparin is preferred as an anticoagulant for plasma, EDTA is widely used and preferable for MS-based metabolomics (whereas NMR cannot use EDTA preserved blood). Proteins should be precipitated and removed unless they are of particular interest. Protein precipitation should not be harsh or abrupt since some of the smaller molecules associated with proteins may co-precipitate and be non-recoverable. Unlike urine, serum and plasma contain high levels of enzymes, if possible, they should be put on ice during extraction. The 4:1 v/v addition of ice-cold methanol (or adding some volume of chloroform to extract nonpolar compounds) to the sample is preferred [23]. It is good practice to work in the dark and degas the extraction solvent to avoid oxidation during this process. The final sample concentration should be high enough to allow sufficient column loading with low injection volume for GC-MS and nano LC-MS analysis. Vacuum centrifugation (Speed-Vac) is a good technique for evaporating organic solvents, but it is better not to evaporate samples to total dryness; this will help to avoid analyte loss and forming a cake-like layer during sample reconstitution. It is recommended to use Freeze-Dryer for lyophilization of the deep frozen aqueous samples, especially for phosphates preservation. For urine samples, lyophilization is needed for concentration if neat urine samples are too diluted for direct analysis.

LC-MS analysis routinely performed with a linear ion trap (LIT) MS operated in full scan mode featuring constant positive and negative mode switching. LC-MS tuning is known to be an important aspect to achieving better sensitivity and ionization efficiency [24,25]. Untargeted metabolic profiling requires a single general tune file for the whole full scan run. We earlier reported [24] significant differences when using five different tuning compounds; sucrose, rutin, naringin, indoleacetic acid, and chlorogenic acid (data not shown). Small molecules are more sensitive to these different tune files than larger polar lipids. Our observations suggested that sucrose should be considered the most suitable tuning compound for MS instrumentation because it provides better responses for most metabolites in both positive and negative ESI modes. Therefore, MS is tuned on a sucrose solution (0.1 mg/mL) in a mixture of acetonitrile and 1g/L ammonium acetate buffer pH 5.5 (1:1, v/v) prior to measurements. It is generally accepted that LC-ESI-MS mass spectra libraries are difficult for standardization; therefore, LC–ESI–MS data are not annotated. Feature LC-MS peaks are subjected to *de novo* identification using LC-Orbitrap or LC-FT-ICR MS for accurate mass measurement, isotopic abundance pattern, MS/MS fragmentation and MS^n^ ion tree experiment, and/or FT-NMR for spatial structural elucidation. Typically, pooled samples are used for *de novo* identification under the same instrumentation conditions. Pooled samples inserted within a batch/group may also serve for quality control purposes during data normalization process.

In addition to deproteinization and lyophilization, samples for GC-MS require a derivatization step to improve the volatility of high-boiling-point analytes. Two-step derivatization is generally applied in metabolomics studies: the first step of methoximation protects ketone and aldehyde groups from reaction in the second step of trimethylsilylation. Freeze-drying for 1–2 hours after solvents removal is important to keep water from the derivatization step because the reactant MSTFA will degrade and generate polysiloxanes. Samples should be injected within 2–24 hours after derivatization because additional time of cold storage results in condensation. Many people also use tertbutyldimethylsil (TBS) derivatization, but may face different challenges. GC–TOF–MS data are annotated using in-house BinBase developed by Dr. Fiehn’s group. Dr. Fiehn and his group deserve the credit on automating the GC-MS annotation procedure so that data are generated as an easy-to-understand excel table [26,27]. Analyte spectra are de-convoluted and aligned in ChromaTOF, identified based on retention index and spectrum similarity match and other additional filters in BinBase. Unique fragment ions for each individual metabolite are chosen as quantifiers and manually corrected when necessary. All known artifact peaks caused by column bleeding, phthalates, or polysiloxanes (typical peaks: *m/z* 221 and *m/z* 281) derived from MSTFA hydrolysis are manually identified and removed from the results.

For large scale metabolomics studies, quality control and assurance (QC/QA) are mandatory [28]. All samples are injected in three independent batches with each batch having a different or totally random injection orders that are automatically generated using in house made software SetupX. Before the batch starts, a performance evaluation mixture (PEM) (sometimes PEM can be substituted by a mid-point standard mix) is used to check the status of the injector, the column, and the mass spectrometer. A reagent blank and a laboratory controlled sample (LCS) are analyzed to test for possible contamination during sample extraction. A five-point calibration curve from a series of dilutions of spiked standard mixes in pooled plasma or urine is analyzed to check the linearity of the MS detector. Sometimes a third-party or project-specific pooled plasma or urine sample is included to compare accuracy of intra- and inter-laboratory results. These QC samples are typically at the beginning and the end of each batch and bracketing every 10 samples within the batch.

Signal intensity, peak shape (width and height), retention time, separation resolution, mass accuracy, and the amount of detectable peaks are monitored using QC samples. After accumulation of enough datasets (typically 20 batches), control charts are established to determine intervention limits. If QC samples fail the intervention limits due to sample preparation errors and/or instrumentation drifting, preventive maintenance (PM) is required. For LC-MS, routine maintenance includes cleaning and flushing column, ion source, sample cones and skimmer, and changing pump oil. For GC-MS, routine maintenance requires changing the septum, inlet liner, gold seal, trimming the first 10 cm of GC guard column, and sometimes may include changing the carrier gas trap, ion source filaments, pump oil, and pump air filter. Instrument tuning (mass calibration and sensitivity check), leak checking, and priming are routinely required after such maintenance and on a scheduled basis.

### 1.3. Data Mining Techniques

Metabolomics datasets require robust data mining technologies [29,30]. Before data mining, results are transformed by total raw normalization, *i.e.*; dividing each peak value (peak area for LC-MS and peak height for GC-MS) by the corresponding metabolite sum and multiplying by a constant factor to eliminate decimal points. Data normalization plays a critical role in urine samples where interpersonal and intrapersonal variances are large. Applying different normalization approaches may help further analysis. Typical normalization protocols are presented in web based MetaboAnalyst package [31].

Unsupervised methods are used to investigate underlying data structure, unbiased by prior knowledge of the experimental design. Principal component analysis (PCA), are well-established techniques for dimensionality reduction and visualization, where the extracted information is represented by a set of new variables, termed as components or vectors [32]. PCA scree plot is considered as a quick quality check and should have a steep decreasing curve with the increase of the Eigen values (meaning that the first several vectors represent majority of the total variance so that visualization process loses minimal information), 3D scatter plot is used to visualize phenotype clusters, and loading plot shows the impact of variables on each vector (the further away from the coordinate center, the more influence).

Visualization methods often are the best way to discover phenotype clusters, whereas clustering methods provide mathematical rigor. Basically, there are three major categories of clustering methods: partitioning (clusters), hierarchical (trees), or probability model-based (models). K-means is the most popular partitioning method, although it requires the input of an initial clustering number. Hierarchical methods construct a binary tree in which the root is a single cluster containing only one element and the leaves each contains only one element. Recently, probability model-based clustering methods have become increasingly popular, with the advances in methods, software, and interpretability of the results.

In order to select prominent potential biomarkers among all the peaks, supervised methodologies with built-in preprocessing and feature selection are needed [33,34,35]. Feature selection is a technique commonly used in machine learning to select a subset of relevant feature for building robust learning models [36]. There are two major categories of feature selection, univariate and multivariate methods. Univariate methods test one metabolite at a time for its ability to discriminate as a dependent variable, and then the most significant metabolites are used to develop a statistical model. Variable ranking, an univariate approach, is widely used because of its simplicity, scalability, and good empirical success. Multivariate methods take into consideration the synergy among metabolites [37,38]. Multivariate search strategies include best-first, branch-and-bound, simulated annealing, and genetic algorithms (GA), using a validation set or cross-validation to assess performance.

GA is a good feature selection algorithm because it automatically selects a small number of feature metabolites during evolutionary learning process and it easily constructs an optimal prediction model with a small number of feature metabolites. It was reported [39,40] that GA is a promising multivariate approach in analysis of the LC–ESI–MS metabolomics datasets. However, GA requires intensive computation and may over-fit data [41]. If the fitness on the training data is significantly better than the fitness on the test data, it indicates over-fitting. In order to avoid over fitting, parameters have to be optimized and independent datasets are needed to validate selected predictors [41,42]. We found that both GA and manual feature selection approaches were able to find different feature sets for differentiation in urine samples of kidney cancer patients. Therefore, we recommend comprehensive feature selection using different approaches and combining the final results of feature components into one set.

### 1.4. Annotation and Identification

Feature components are considered as potential biomarkers and are used for annotation and *de novo* structural identification [43]. Annotation of the MS and MS/MS spectra is done using commercial NIST05/Wiley Registry, METLIN [44], MassBank [45], Human Metabolome Database [46], Lipid Maps [47], BinBase [48], KEGG [49], and in-house mass spectral libraries. Annotation is further validated with *de novo* structural identification.

Feature components are analyzed in SIM mode on a high-resolution high-accuracy MS such as LTQ Orbitrap or LTQ FT Ultra coupled with an LC for accurate mass measurement. We found that LTQ-Orbitrap MS is 10–100 times more sensitive than LTQ-FT Ultra MS, but the latter can go to the resolution of 1,000,000 at *m/z* 400 and has better mass accuracy (routinely reached to the range of 0.1–2 ppm without any lock mass or reference mass, to ppb level mass accuracy when using an internal mass calibration standard). Therefore, LTQ-Obitrap MS is used to identify low abundance unknown metabolites, whereas LTQ-FT Ultra MS is used when maximum mass accuracy and resolution are needed. Although it is very expensive, requires heavy maintenance, and needs extensive optimization, in our opinion, LTQ-FT Ultra MS is the best instrument for element composition analysis due to its extreme accurate mass measurement. LC coupling to LTQ FT Ultra MS can only go to MS^2^ and the fragmentation spectra are unit mass resolution, because MS/MS fragmentation occurs in the LTQ ion trap but not in the FT-ICR cell. Accurate mass ion tree spectra up to the MS^10^ can be obtained using Nanomate nano-ESI robot coupled with LTQ FT Ultra MS. Accurate mass ion tree experiment is only possible using direct injection nano-ESI, due to the long scanning time required for the FT-ICR cell to do MS^n^ experiment. Using infrared multiphoton photodissociation (IRMPD) or other FT-ICR cell specific fragmentation mechanisms, isolation of parent ions is also high resolution and high accurate mass so that total (ions of parent and daughter and further generations) accurate mass ion tree is possible.

In order for LTQ-FT Ultra MS to work with MassWorks^TM^, the following parameters need to be adjusted. (a) In LTQ-FT Ultra MS, the Active Noise Reduction option in Instrumental Configuration_FT settings should be unchecked because Active Noise Reduction uses an advanced algorithm to decrease the statistical noise in an FT spectrum without increasing the noise threshold. If the Active Noise Reduction is turned on the data size of acquired raw files is significantly decreased, therefore useful data for later processing may be lost. (b) Another option of AGC_Enable Full Scan Injection Waveforms should be unchecked. This option is to apply a filter on the injection ions. The ions above and below the selected ion or ion range selected are rejected. This option is often useful if the ion trap is being filled with ions of greater or lesser mass than the ion mass or ion mass range of interest. In the modes of FT-SIM, FT-MS^n^, FT-ECD, and FT-IRMPD, the injection waveforms are automatically enabled. (c) Automatic gain control (AGC) should be used to set the ion injection time to maintain the optimum quantity of ions for each scan. With AGC on, the scan function consists of a prescan and an analytical scan. The MS detector measures the flux of incoming ions for the prescan. This information allows the MS detector to determine the optimum ion injection time for the analytical scan. The ion injection time information is then used to scale the resulting values obtained by the analytical scan. Therefore, AGC is used to extend the dynamic range of the MS detector beyond its fundamental dynamic range.

The data acquired with high resolution MS are insufficient for assigning unique elemental compositions without supporting information on isotope ratios [50,51]. The isotopic abundance pattern serves as an additional powerful constraint for identification of candidates with very similar elemental composition. Previous studies demonstrated that interpretation of isotopic abundance patterns can remove more than 95% of false candidate formulas for molecules below 500 Da [50,51]. Studies concluded that instruments with 3 ppm mass accuracy and 2% relative error for isotopic abundance pattern outperform those with less than 1 ppm accuracy but without including isotope information in the calculation of molecular formulas. Self-Calibrated Lineshape Isotope Profile Search (sCLIPS) algorithms correct the instrument’s line-shape and enable exact isotope modeling when comparing the MS response of an unknown ion against theoretically calculated responses for all possible candidate formulas and data acquired in continuous mode. As long as the isotopic pattern can be measured accurately, sCLIPS algorithms can be used to predict elemental composition of any molecule. Actually, our recent experimental data supported the idea of using exact isotope modeling as a key filter for unique elemental formula assignment [52].

Mass spectra of potential biomarkers obtained with a high-resolution high-accuracy MS are spectrally corrected using sCLIPS in MassWorks^TM^ to achieve high mass and spectral accuracy after data acquisition. A good, free alternative is the “Seven Golden Rules” software package designed by Drs. Tobias Kind and Oliver Fiehn [50,51]. The unique elemental formula is searched against CAS database using the strategy of Explore Substances—Chemical Structure for known compounds, or input into the MolGen 3.5, thus generating all of the possible structural isomers corresponding to the elemental formula. The chemical structures are saved and imported to Mass Frontier^TM^ for MS^n^ fragmentation modeling analysis. The Mass Frontier^TM^ Fragments and Mechanisms module is an expert system providing information about basic fragmentation and rearrangement processes based on literature, starting from a user-supplied chemical structure. The theoretical fragments generated by Mass Frontier^TM^ are compared to those acquired from FT-ICR MS. Parent compounds with the best match of MS^n^ fragmentation pattern are considered as the molecular structures of the potential biomarkers. The latest version of Mass Frontier^TM^ enables one to build up a library of accurate mass ion tree spectra so that unknown identification is even more reliable.

For validation purpose, the proposed molecules are searched against chemical structure and property databases or search engines; such as, PubChem [53], Chemical Structure Lookup Service (CSLS) [54], CRC Dictionary of Natural Products (DNP) [55], ChemSpider [56] , and/or proprietary Beilstein Database using MDL Crossfire Commander and Chemical Abstracts Database (CAS) using SciFinder Scholar.

In addition to FT-ICR MS, in some complicated cases, NMR is needed for structural elucidation of novel metabolites for which nothing is known [57,58]. LC fractions using semi-preparative columns are collected automatically by a fraction collector, further concentrated by freeze-drying, then reconstituted in D_2_O (hydrophilic fractions) or deuterated methanol/chloroform (lipid fractions). For D_2_O fraction, DCl and NaOD solutions are used to adjust pH to neutrality. Each fraction is then analyzed by 1D and 2D high resolution NMR. NMR raw data are apodized, Fourier transformed, phase and baseline corrected. From 1D ^1^H and ^13^C NMR spectra, relative numbers and types of hydrogen and carbon atoms can be determined. For 2D NMR homonuclear experiments, correlation spectroscopy (COSY) and double quantum filter DQF-COSY detect connections between protons coupled to each other with bonding (^1^*J*_HH_, ^2^*J*_HH_, ^3^*J*_HH_, ^4^*J*_HH_), total correlation spectroscopy (TOCSY) provides information of all coupling protons with bonding correlation in a given spin system; whereas nuclear overhauser effect spectroscopy (NOESY) and rotating overhauser effect spectroscopy (ROESY) establish interaction of non-bonding protons with a distance up to 4 A (^1^H-^1^H). For 2D NMR heteronuclear experiments, gradient enhanced (*ge*) variants (with better sensitivity) of heteronuclear single quantum correlation (HSQC) and heteronuclear multiple quantum correlation (HMQC) find correlation between a proton and a carbon connected with a bond (^1^*J*_CH_), whereas, 2D heteronuclear multiple bond correlation (HMBC) and ^2^*J*_CH_,^3^*J*_CH_-HMBC NMR spectra are more sensitive to ^2^*J*_CH_ and ^3^*J*_CH_. Amazingly, 3D HMQC-COSY and HMQC-TOCSY experiments are able to determine *J*_HH_ and *J*_CH_ simultaneously so that ^13^C signals can be easily assigned.

### 1.5. Predictive MRM Screening of Secondary Metabolites.

After identification of the feature compounds, their potential secondary metabolites are screened using the Predictive Multiple Reaction Monitoring (pMRM) mode available on triple quadruple-linear ion trap mass spectrometer (QTRAP). Currently, the analysis of low-abundant metabolites remains an unresolved problem in metabolic profiling. In spite of being able to detect many metabolites, neither TOF MS nor ion trap MS performing in full scan mode, is sensitive enough to detect and characterize metabolites at trace levels. Triple-quadruple (QQQ) tandem mass spectrometer (MS/MS) provides excellent sensitivity in multiple reactions monitoring (MRM) mode, but lack structural information and metabolite coverage.

Recently, a hybrid QTRAP MS system combining a triple-quadruple scanning functionality with sensitive LIT scans is commercially available. Working in LIT mode, the QTRAP MS provides improved performance with enhanced sensitivity in enhanced full scan (EMS) and enhanced product ion scan (EPI) modes. Additionally, the instrument can be operated under all the triple-quadruple scanning modes including MRM, as well as, precursor ion and constant neutral loss scan. Therefore, accurate quantification and additional structural information can be obtained simultaneously in a single run by combing MRM and EPI scanning via the built-in information-dependent acquisition (IDA) functionality.

pMRM-IDA-EPI has been used in our group [43,59,60,61,62] and in drug discovery [63,64,65,66,67]. The pMRM-IDA-EPI method is composed of one MRM scan, one IDA criteria, and one enhanced product ion (EPI) scan. The pMRM algorithms generate theoretical metabolite MRMs based on the latest updated database composed of more than 100 well-known Phase I and Phase II biotransformations or customized transformations reflecting endogenous metabolic pathways [68]. More interestingly, the pMRM approach can identify positional isomers which are helpful in identification of positional changes in metabolism. For example, clomazone has a molecular ion [M+H]^+^ mass charge ratio of *m/z* 240, and the fingerprint fragment is *m/z* 125, the phenyl ring substructure. Di-oxidation adds two oxygen atoms on the parent molecule. After the collision of the parent ion in Q_2_, the fingerprint fragment may be *m/z* 125 + 2 × 16, *m/z* 125 + 1 × 16, and/or *m/z* 125, corresponding to di-OH-clomazone isomers with 2, 1, and 0 oxygen atoms on the phenyl ring substructure of the clomazone [43,59,60,61].

Although the pMRM approach is good at finding compounds at trace levels due to the high sensitivity of QTRAP MS in pMRM mode, the limitation of unit resolution and low mass accuracy warrants further validation using a high-resolution high-accuracy MS. Unfortunately, some metabolites detected in pMRM mode are not detectable with any other modes of acquisition on a QTRAP, an LTQ-Orbitrap, or an LTQ-FT Ultra MS. On the other hand, this actually demonstrates the high selectivity and sensitivity of pMRM mode. Therefore, in order to identify these metabolites, preparative HPLC fractionation and concentration are necessary, and offline spatial structural elucidation with NMR and accurate mass measurement with a high resolution MS are needed to confirm proposed structures.

### 1.6. Pathway Analysis

Metabolic pathway analysis is needed to understand whether feature metabolites/potential biomarkers identified from comprehensive metabolomics studies are the key metabolites involved in the altered metabolic pathways; resulting in disease-induced metabolome changes. It is difficult to interpret these observed changes *in vivo* due to compartmentalization. However, these impacted pathways may be considered as biomarkers themselves, including all of the intermediate metabolites, proteins, and genes involved. Ideally, metabolic flux studies using stable isotope tracers are more suitable for metabolic pathway analysis. It is known that the pool size of a compound is not equal to its kinetics flux rate in human body. In order to know the flux rate of a compound, stable isotope trace studies are needed. Classic flux studies focus on small scale known network assessment and are performed mostly on cultured cells. Metabolomics studies can provide valuable information for large-scale unbiased pathway analysis, with certain limitations [69]. First, extraction protocols in metabolomics studies do not involve separation of cellular sub-organelles; therefore, results from metabolomics studies lack information of subcellular compartments. Second, metabolomics studies typically lack kinetic experimental design to address the issue of multiple steady-states, so that results are difficult to interpret even when pool sizes are measured. Third, *de novo* identification is required to elucidate the structure of unknown metabolites, which is a daunting task.

Nevertheless, time-course snap-shots of metabolome have been shown to be effective in network construction [69]. Metabolite pathway analysis use comprehensive pair-wise metabolite correlations and metabolite co-response coefficients, assuming intermediate metabolites in specific pathways change in a correlated way. Each node represents a certain metabolite, the more connections of this node to other nodes, the more important it is in the network. Typically, data needs to be log-scaled to minimize the impact of outliers on final pathway construction and the network graph layout uses special software packages and algorithms such as Pajek.

Another approach is to see the overlap between the feature metabolites and a database of metabolites associated with various biological pathways; the more overlapping between the two, the more impact the feature metabolites on this/these specific pathway(s). The database can be assembled from manually collected and curated pathway maps from previous literature. Open-access pathway database KEGG can be used with web-based MetPA [70]. Another pathway database BioCarta is accessed and analyzed using the commercial software package Ingenuity Pathway Analysis [71]. MetPA uses KEGG database to search pathways, KEGGgraph to parse pathway topology into graph models, and Graphviz and ImageMgick to manipulate graphs. Based on the centrality measures of a metabolite, MetPA is able to estimate a node’s relative importance in a given metabolic network.

The third approach, of unbiased network construction, is to apply text mining techniques searching through public literature repositories (such as PubMed) so that a network is built based on putative associations suggested in the text of the articles. This approach does not need already-built network databases. The advantage of this approach is that it utilizes previous knowledge base without *a priori* assumptions and is very useful at generating hypothesis for further experiments. A free open-access software package, Cytoscape, is well accepted for this purpose [72,73]. Basically, a list of identified potential biomarkers is entered into Cytoscape and high-scoring network clusters (complexes) and genes with the highest connectivity (seeds) are identified. Seed genes are further searched in Cytoscape to construct networks containing known and putative functional associations between the genes of interest. Finally, the biological functions of seed genes are summarized automatically.

## 2. The Application in Early Diagnosis of Pancreatic Cancer

### 2.1. Introduction

Pancreatic ductal adenocarcinoma (PDAC) ranks fourth as the cause of cancer-related mortality and second among the gastrointestinal cancers in the USA [74,75,76]. Surgery is the main curative treatment modality, however, most patients present at a late stage when the curative intervention is not available, as surgical resection is performed only in 10% to 15% of such patients [77]. Furthermore, surgical treatment failures occur due to local or metastatic recurrences, commonly presenting within one to two years of the index operation [78]. With the high contribution of late-stage discovery and present unavailability of effective medical treatment, the key approaches in improving the poor outcome of pancreatic cancer is to focus on early detection of the tumor. Currently, there is no available biomarker with good enough sensitivity and specificity for early detection of pancreatic cancer. In this case study with previously published data, the workflow of untargeted metabolomics is described to discover novel biomarkers of early pancreatic cancer [73].

### 2.2. Procedure

#### 2.2.1. Material

Oligosaccharides kit, methoxylamine hydrochloride, pyridine, N-methyl-N-trimethylsilyltrifluoroacetamide (MSTFA) and reserpine were all purchased from Sigma-Aldrich (St. Louis, MO, USA). Ammonium acetate, ammonium hydroxide, and acetic acid were the highest purity grade available from Sigma-Aldrich. Extra pure formic acid was purchased from Fluka (Sigma-Adrich, St. Louis, MO, USA). LC–MS grade acetonitrile, methanol, and water were purchased from Burdick and Jackson (VWR International, West Chester, PA, USA). Purity of each lot was investigated by LC-MS infusion. Fresh aqueous buffers for LC-MS were prepared on the working day. Reserpine stock solution was 0.2 mg/mL in methanol. MSTFA, pyridine, and/or mixed reagents were stored under dry nitrogen after opening the bottle/ampoule or preparing the mix. The storage was in aliquot with tight seal. Each lot of organic solvents was investigated by LC/MS analysis.

#### 2.2.2. Sample Preparation

After plasma was thawed on ice, 400 µL of ice-cooled methanol was added to 100 µL of plasma in a 2-mL microfuge tube. After 3 pre-freezed metal balls (one 3mM i.d. and two 2mM i.d.) were added, the mixture was homogenized (30 cycles per second × 2 min) in a pre-chilled tube holder (–80 °C × 1 h) in Retsch Ball-Mill (Newtown, PA, USA). Then, the mixture was sonicated in an ultrasonic bath (ambient temperature × 1 min), extracted in dark on an orbital shaker (Torrey Pines Scientific Inc.; San Marcos, CA, USA) (750 rpm at 4 °C × 2 h), centrifuged at 4 °C (13,000 rpm × 5 min). The supernatant was transferred to a fresh 2-mL microfuge tube, and dried in Speed-Vac and Freeze-Dryer (Labconco, Kansas City, Missouri, USA). For GC-MS, dried samples were used for derivatization. For LC-MS analysis, dried samples were reconstituted in 100 µL water-acetonitrile (1:1, v/v), the clear supernatant was transferred to a HPLC vial (MicroSolv Technology, Eatontown, NJ) with an insert and a pre-slit cap (MicroSolv).

#### 2.2.3. Untargeted Metabolic Profiling

##### 2.2.3.1. LC-MS

The LC-MS system consisted of an ACQUITY UPLC system composed of a binary solvent manager, a sample manager, a column manager, and a TUV detector (Waters Corp.; Milford, MA, USA), with a working backpressure of 10,000 psi, coupled to a LTQ (Thermo Fisher, San Jose, CA, USA) linear ion trap (LIT) mass spectrometer operated under Xcalibur software (v1.4, Thermo Fisher) without splitting. Injection volume was set at 10 μL. The entire effluent from the HPLC column was directed into the electrospray ionization source (ESI) of the LTQ MS. Normal flow HPLC (0.3–1 mL/min) with conventional or microbore columns required pneumatically assisted ESI. The ESI ion source was equipped with a metal needle. The electrospray voltage was set to 5 kV. Nitrogen sheath and auxiliary gas flow rates were set at 60 and 20 arbitrary units, respectively. The ion transfer capillary temperature was set at 350 °C with typical ion gauge pressure of 0.90 × 10^−5^. Full scan spectra were acquired from 100–1,000 amu at unit mass resolution with maximum injection time set to 200 ms in one micro scan. Acquisition was performed in both positive and negative continuous switching modes. A sucrose tune file in negative/positive modes at normal LC flow rate was used. For MS^n^ experiments, data dependent scans were chosen with the wideband activation turned off. The normalized collision energy was set to 35%, the activation time to 30 ms, and the activation Q to 0.250, with the source fragmentation turned off.

###### 2.2.3.1.1. RP ESI-LC-MS

For human blood plasma samples, RP-LC-MS was performed on a BEH C8 shielded Acquity UPLC column (150 × 2 mm, 1.7 µM particle size, Waters). Analytical liquid chromatography was performed using 1 g/L ammonium acetate (pH 5.5, adjusted by glacial acetic acid) (A) and acetonitrile/acetone mixture (9:1, v:v) (B) as the mobile phases with a column temperature of 70 °C and a flow rate of 0.5 mL/min. After a 0.1 min isocratic run at 1% B, a sequential ramping up to 100% B was followed for total elution time of 10 min. Then, at 100% B LC was held for another 5 min to wash the column off non-polar residues, going back to 1% B in 1 min and held for another 5 min to equilibrate the column with the initial mobile phase composition. To avoid contaminating MS, the divert valve was opened to the waste line during first several minutes of solvent peak elution and during column washing and equilibration. Weak wash solvent was H_2_O-methanol (1:1, v/v) and strong wash solvent was acetonitrile/isopropanol (3:1, v/v). Reserpine was used as an external standard for instrument calibration and semi-quantitative analysis. A series of dilutions of reserpine stock solution in methanol was prepared starting from 0.1 mg/mL. Five or more points were acceptable for creating a calibration curve of reserpine. In addition, reserpine was also spiked in samples as internal standard for QC purpose.

###### 2.2.3.1.2. HILIC ESI-LC-MS

For human blood plasma samples, HILIC-LC-MS was performed on a Luna HILIC Diol HPLC column (150 × 3 mM, 3 µM particle size, Phenomenex, Torrance, CA, USA). The mobile phases were 100 mM ammonium formate (pH 4.0) (A) and acetonitrile (B) with a column temperature of 40 °C and a flow rate of 0.4 mL/min. After a 2-min isocratic run at 3% A, sequential ramping scheme was followed up to 40% A for a total injection time of 20 min. Then, the column wash was done with 100% A for 5 min. Column equilibration with initial mobile phase composition took 15 min before the next injection. The weak wash solvent was acetonitrile-isopropanol (3:1, v/v) and the strong wash solvent is H_2_O-methanol (1:1, v/v). The divert valve was opened to the waste line during first several minutes of solvent peak elution and during column washing and equilibration.

For HILIC–LC–ESI–MS analysis, oligosaccharides were used as retention time index standards because they eluted in the order of increasing monomer units, with larger oligomers eluting as the latest ones. Mono and oligosaccharides were detected as ammonia adducts in positive mode and as [M-H]^−^ ions in negative mode. The Sigma-Aldrich oligosaccharides kit was prepared in acetonitrile:water (1:1, v/v). Total concentration did not exceed 0.5 mg/mL. Then, selected oligomers were used as internal and/or external standards for instrument calibration by serial dilution.

##### 2.2.3.2. GC–MS

GC–TOF-MS analysis was performed using an Agilent 6890 N gas chromatograph (Atlanta, GA, USA) interfaced to a time-of-flight (TOF) Pegasus III mass spectrometer (Leco, St. Joseph, MI, USA). Automated injections are performed with a programmable robotic Gerstel MPS2 multipurpose sampler (Mülheim an der Ruhr, Germany). The GC was fitted with both an Agilent injector and a Gerstel temperature-programmed injector, cooled injection system (model CIS 4), with a Peltier cooling source. An automated liner exchange (ALEX) designed by Gerstel was used to eliminate cross-contamination from sample matrix occurring between sample runs. Multiple baffled liners for the GC inlet were deactivated with 1-µL injections of MSTFA. Initial peak detection and mass spectrum deconvolution were performed with ChromaTOF software (version 2.25, Leco), and later files were exported to the netCDF format for further data evaluation.

After the extracts were completely dried by speed vacuum concentrator or by freeze-drying, 20 µL of 40 mg/mL methoxylamine hydrochloride in pyridine was added, and samples were agitated at 30 °C for 30 min. Subsequently, 180 µL of trimethylsilylating agent, N-methyl-N-trimethylsilyltrifluoroacetamide (MSTFA), was added, and samples were agitated at 37 °C for 30 min. A mixture of the retention time standards, n-dodecane (RI 1200), n-pentadecane (RI 1500), n-nonadecane (RI 1900), n-docosane (RI 2200), n-octacosane (RI 2800), n-dotriacontane (RI 3200), and n-hexatriacontane (RI 3600) was included in the final reagent volume. Analytical GC chromatography was performed with the injection volume of 1 µL with a split ratio of 1:10 (purge time 120 sec, purge flow 40 mL/min). The Agilent injector temperature was held constant at 250 °C while the Gerstel injector was programmed (initial temperature 50 °C, hold time 0.1 min, and increased at a rate of 10 °C/s to a final temperature of 330 °C, hold time 10 min). Chromatography was performed on an Rtx-5Sil MS column (30 m × 0.25 mM i.d.; 0.25 µM film thickness) with an Integra-Guard column (Restek, Bellefonte, PA, USA). Helium carrier gas was used at a constant flow of 1 mL/min. The GC oven temperature program was 50 °C for initial temperature with a 1-min hold time and ramping at 20 °C/min to a final temperature of 330 °C, with a 5-min hold time. Both the transfer line and source temperatures were 250 °C. After a solvent delay of 350 sec, mass spectra were acquired at 20 scans per second with a mass range of 50 to 500 m/z. Ion source filament energy was set to 70 eV.

#### 2.2.4. Data Mining

##### 2.2.4.1. Free Open Source Software

For LC-MS datasets, free R-based XCMS and Java-based Mzmine were used for pre-processing in our group. Because Mzmine took almost one week analyzing a relatively small dataset, XCMS was preferred. The Xconvert program included in Xcalibur was used to convert the Xcalibur (*.raw) files to netCDF (*.cdf) format. A free software package msconvert can also do format converting [79]. Automatic peak finding, de-convolution, and alignment were performed using XCMS. XCMS parameters were: group bandwidth, 30, minimum fraction, 0.5, minimum sample parameter, 1, width of overlapping *m/z* values, 0.5, maximum number of groups in a single *m/z* slice, 0.5. After processing and peak picking, mass spectral features were retrieved from XCMS as a TXT file. Total raw optimization was applied because it was shown to be useful for serum and urine samples. Peaks were normalized to the total absolute area of all detected metabolites in each sample using an in-house written R script.

Preliminary data exploration was accomplished using unsupervised methods such as principle component analysis (PCA) and clustering. For PCA, R package pcaMethods in Bioconductor project was used to generate a scree plot (to show the optimal number of eigenvalues), a score plot (to show the most important principal components and visually detect clusters), and a loading plot (to show positive and negative correlations of components). Cluster analysis of the PCA scores was performed using partitioning methods such as K-means using the function kmeans in R package stats, hierarchical agglomerative methods such as Ward's method using the function hclust in R package stats, and multiscale bootstrap resampling using R package pvclust, and model-based clustering approach using R package mclust which assumes a variety of data models and applying maximum likelihood estimation and Bayes criteria to identify the most likely model and number of clusters.

Feature selection using GA procedure and further classification were performed using R package GALGO. With hardware and software currently used, two days were needed for one run using GA with nearest centroid method. The GA parameters were optimized: the nearest centroid classification method was applied, the maximum solutions value was set as 2,000 to stabilize top 20 feature components, the maximum generations value as 500 because thousands of generations end up in over-fitting, the goal fitness as 1.0, the subset (chromosome) size as 5, the population size as 20 plus an additional unit per each 400 variables, the mutation rate as 1 mutation per subset (chromosome), and the crossover value as all subsets (chromosomes) in exchange.

For GC-MS dataset, peak finding was done in ChromaTOF, and the peak table was exported to BinBase for annotation. In ChromaTOF, a peak-finding data-processing method was created with the activation of the following functions: “Retention Index”, “Peak Find”, “Baseline”, and “Calculate Area”. Key parameters were optimized as following: baseline offset as baseline subtraction just above the noise level, data points to be averaged for smoothing as no smoothing, expected chromatographic peak width as 3 sec, and minimum signal-to-noise ratio for the automatically chosen quantitation mass as 10:1. The apex masses, complete spectrum, retention time, peak purity, noise, signal-to-noise ratio, unique ion, and unique ion signal-to-noise ratio of each peak were exported as txt file. This txt file was further processed in BinBase with following parameters: validity of chromatogram (< 10 peaks with intensity > 10 million), unbiased rention index marker detection (MS similarity > 800, validity of intensity range for high m/z marker ions), retention index calculation (5th order polynomial regression), spectral validity (> 5% of base peak abundance, matching least and most-abundant spectra in database using the following matching filters: retention index window ± 2,000 U / ± 2 sec, unique ion in apex masses and > 3% of base peak abundance, mass spectrum similarity 500 if s/n > 25 and purity >1.5).

##### 2.2.4.2. Commercial Software

Compared to FOSS workflow, commercial software packages have user-friendly interface and need less time for optimization, at the expense of high cost and undisclosed algorithms involved in calculation. Prior to data processing, original Xcalibur LC-MS files (*.raw) were converted to netCDF (*.cdf) format using the XConverter (Thermo Fisher) or msconvert, then converted to WIFF format using Analyst QS for use in MarkerView^TM^ software (version 1.1, Applied Biosystems, Foster City, CA, USA). For RP and HILIC LC-MS data, peak finding options were set as follows: subtraction offset, 10 scans; subtraction multiplication factor, 1.3; noise threshold, 3; minimum spectral peak width, 1 amu; minimum retention time peak width, 4 scans; and maximum retention time width, 1000 scans. Peak alignment options were set as follows: retention time tolerance, 0.5 min; mass tolerance, 0.8 amu; and maximum number of peaks, 5000. Peaks found in fewer than 6 of the samples were discarded using filter setting. Peak area integration was performed using raw data. Peaks were then normalized to the total absolute area of all detected metabolites in each sample.

The data that constituted retention time, mass to charge ratio, and peak areas of detected and aligned peaks were exported from MarkerView^TM^ into Statistica^TM^ version 9 [80] for principal component and classification analysis (PCCA). The analysis was carried out via the correlation matrix, or the covariance matrix of the standardized (scaled) variables. Univariate feature selection and one-way analysis of variance (ANOVA) were conducted after PCCA. For continuous predictors, the range of values in each predictor was divided into 10 intervals, whereas categorical predictors could not be transformed in any way. For categorical dependent variables for classification-type problems, a Chi-square statistic and p-value for each predictor were computed and used as the criteria of predictor importance to select best predictors.

#### 2.2.5. Annotation and Identification of Feature Components

##### 2.2.5.1. Accurate Mass, Isotope Pattern, and MS/MS

A fast approach was used for accurate mass, isotope pattern, and MS/MS acquisition. HPLC method was the same as described earlier. The entire effluent from the HPLC column was directed into the ESI source of an LTQ-Orbitrap (Thermo Fisher Scientific, San Jose, CA) operated under Xcalibur software (V2.07, Thermo Fisher Scientific) or an LTQ-FT Ultra hybrid linear ion trap –7.0 T Fourier transform ion cyclotron resonance (FT-ICR) MS (Thermo Fisher Scientific) operated under Xcalibur software (V2.2, Thermo Fisher Scientific). Both LTQ-Orbitrap and LTQ-FT Ultra MS used identical source and scanning parameters. The ion source voltage was 5 kV. Nitrogen sheath and aux gas flow was 60 and 20 units respectively. Nitrogen was produced by a nitrogen generator system (Peak Scientific, Billerica, MA, USA). The ion transfer capillary temperature was 350 °C. Typical ion gauge pressure was 0.90 × 10^−5^ Torr. Survey (full scan *m/z* with a width of 10 Da) and single ion monitoring (narrow SIM with a width of 10 da) MS spectra were acquired with the resolution R = 50,000 (FWHM) in LTQ-Orbitrap and R = 100,000 (FWHM) in LTQ-FT Ultra at *m/z* 400. All scan events were acquired with one micro scan. Full scan spectra were acquired with a 200 ms maximum ionization time. The AGC target value was set as 1,000,000 in the linear ion trap. In addition, mass spectrometer was operated in the data dependent mode to automatically switch between MS and MS/MS acquisition. The most intense ions were isolated and fragmented in the linear ion trap using collisionally induced dissociation (CID) at a target value of 100,000. Parameters applied in MS/MS scan events were an isolation width of 2 Da, an activation time of 30 ms, normalized collision energy of 40%, and an activation Q of 0.250. Data dependent dynamic exclusion was used with the following parameters: repeat count, 5; repeat duration, 15 s; exclusion duration, 60 s.

Elemental composition was obtained using MassWorks (version 2, Cerno Bioscience, Danbury, CT, USA), XCalibur embedded elemental composition tool (V2.2, Thermo Fisher), and Mass Frontier embedded formula generator tool (version 5.1, HighChem Ltd, Bratislava, Slovakia). Mass spectra of the feature peaks were spectrally corrected using MassWorks to achieve high mass and spectral accuracy after data acquisition, using sCLIPS algorithms enabling exact isotope modeling. Using reserpine as an example, the parameters for MassWorks sCLIPS algorithms were: mass tolerance, 5 ppm; electron state, even; double bond equivalent range, −3 to 50; profile mass range, −1 to 3.5 Da; calibration range, −0.1 to 0.1 Da, element-max, C-48, H-576, O-41, N-41. The parameters for XCalibur embedded elemental composition tool were: mass tolerance, 5 ppm; nitrogen-rule, even electron ions; RDBE (ring plus double bonds equivalent), −3 to 50; element-max, C-48, H-576, O-41, N-41. The parameters for Mass Frontier embedded formula generator tool were: mass tolerance, 5 ppm; nitrogen-rule, even electron ions; RDBE, −3 to 50; element-max, C-48, H-576, O-41, N-41.

##### 2.2.5.2. MS^n^ Ion Tree

Sometimes accurate mass MS^n^ ion tree scans were needed for *de novo* identification. First, feature peaks were purified using semi-preparative LC, collected in fraction collector, lyophilized, reconstituted with 1 g/L ammonium acetate in water-acetonitrile (50:50, v/v), and loaded on wells of a 96-well plate. Nano ESI direct infusion analysis was achieved using a NanoMate nanoESI chip robot (Advion BioSciences, Ithaca, NY, USA) coupled with an LTQ-FT Ultra MS operated under Xcalibur software (V2.2). Nanomate holds a 96-well plate, a rack of 96 disposable conductive pipette tips, and a nanoESI chip consisting of a 20 × 20 array of nozzles etched from the planar surface of a silicon wafer. Using reserpine as an example, 10 μL of sample mixture were delivered from the well on the plate to the back plane of the nano-ESI chip with the optimized positive ionization conditions of 1.65 kV in positive mode and 0.5 psi nitrogen head pressure, so that the solution in the pipette tip had a constant flow to the chip at a rate of 200 nL/min.

The parameters for LTQ-FT Ultra MS were: ion source voltage, 3 kV; nitrogen sheath and aux gas flow, 0 and 0 units, respectively (because nano-ESI uses low volume of solvent and does not require gas-assisted de-solvation and ionization process); ion transfer capillary temperature, 250 °C; capillary voltage, 35 V; tube lens, 130 V; typical ion gauge pressure, 0.60 × 10-5 Torr. The ion tree acquisition method was generated using XCalibur software using default values. All scan events were acquired with one micro scan. Full scan spectra were acquired with a 2,000 ms maximum ionization time. The default AGC target value was used for narrow SIM (50,000 ions) and full scan modes (500,000 ions).

#### 2.2.6. Pathway Analysis

Pathway analysis was performed utilizing MetPA and Ingenuity Pathway Analysis (IPA) . The list for identified metabolites detected in all samples was annotated with common chemical names and submitted into MetPA. The parameters for MetPA were customized as following: library as “Homo sapiens”, metabolome as “All Compounds”, over-representing test as “Hypergeometric Test”, pathway topological analysis using default values of “Relative-Betweenness Centrality”. Verification of accepted metabolites was conducted manually using HMDB, KEGG, and PubChem databases. The metabolite list was also uploaded into the IPA Knowledge Database. Canonical pathway analysis revealed the biological pathways, networks and diseases associated with the identified metabolites.

A list of identified potential biomarkers was also entered into Cytoscape plugin Agilent Literature Search. High-scoring network clusters (complexes) and genes with the highest connectivity (seeds) were identified using MCODE plugin. Seed genes were further searched in cPath plugin to construct networks containing known and putative functional associations between the genes of interest. The biological functions of seed genes were summarized using BiNGO plugin.

### 2.3. Results and Discussion

#### 2.3.1. Experimental Design

In the preliminary analysis of 20 cancer patients and 20 controls, we found that plasma sampling date was a significant confounding factor. Therefore, five case-control pairs were selected with plasma sampling dates controlled within 3 months in each case.

#### 2.3.2. Principal Component Analysis

**Figure 1:**
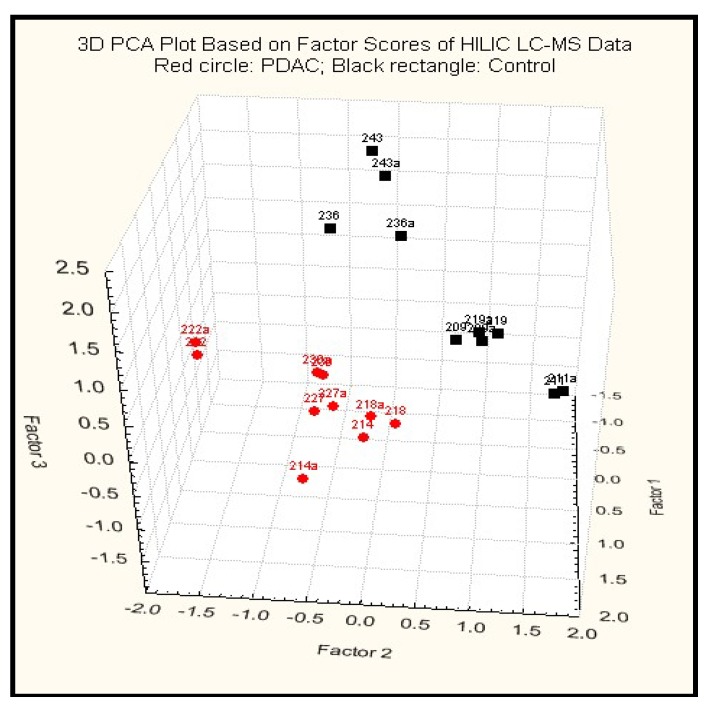
A 3D PCA plot of results using HILIC-LC-ESI-MS.

PCA was used for preliminary data mining on whole set of annotated peaks from GC-MS and not annotated peaks from HILIC and RP LC-MS in an unsupervised fashion. PDAC cases (red) were well separated from noncancerous controls (black) (Figure 1). The first 3 principal components explained 58.24% of total variance, suggesting that these 3-D PCA plots were representative of the original data. HILIC LC-MS dataset was found to have the best separation between groups.

Groups in the right panel were color-coded as: 1—red (PDAC), 2—black (normal & chronic pancreatitis).

#### 2.3.3. Putative Biomarkers

Feature selection was conducted to select the best predictors for PDAC final diagnosis. If the analysis showed significant difference between PDAC patients and controls with satisfactory p value and high *p* variance value, and manual inspection of corresponding extracted mass chromatograms revealed satisfactory peak, the metabolite was chosen for annotation and further *de novo* structural identification and considered as putative biomarker of PDAC (Table 1). What we presented in Table 1 were potential unknown classifiers, requiring further investigation for final structural determination. Comparing the unknown peaks with available authentic standards was done for certain important components. Interestingly, HILIC separation was useful not only in classifiers detection/annotation but also in investigation of authentic standards.

**Figure 2:**
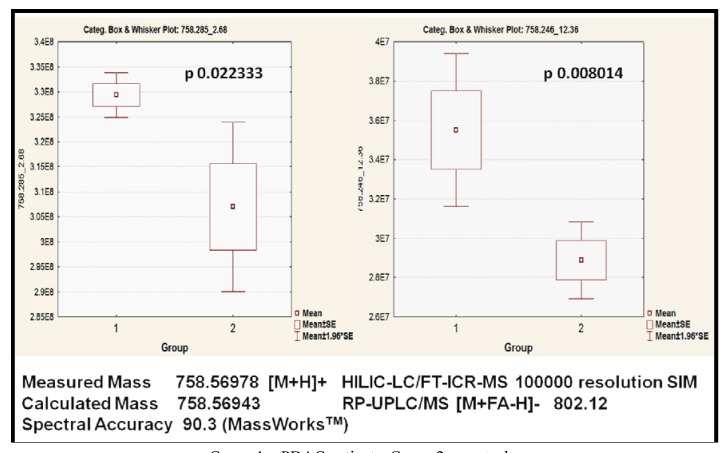
Box-Whisker plots of the potential classifiers at RT 2.68 min (left panel) and RT 12.36 min (right panel), which were later identified as PC (34:2) isomers.

**Figure 3:**
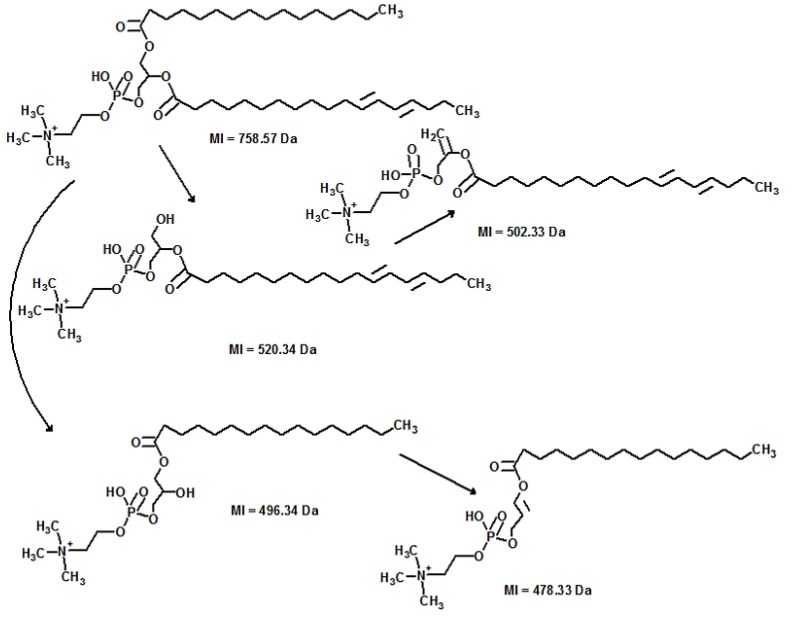
ESI-MS/MS fragmentation pattern typical for all observed isomers of polar lipid PC (34:2) found in this study.

**Figure 4:**
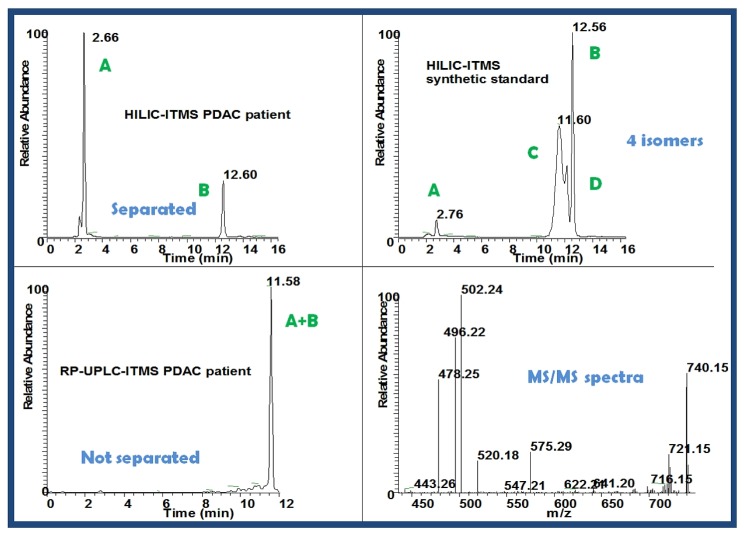
Extracted chromatograms of the positive ion *m/z* 758 and the corresponding MS/MS spectra from the human plasma sample of a PDAC patient and from a commercial authentic standard.

For example, two feature peaks of m/z 758 were found significantly higher in PDAC patients on HILIC LC-MS at retention time of 2.68 min (*p* = 0.022) and 12.36 min (*p* = 0.008) (Figure 2, Figure 4). Accurate mass of the component was measured as 758.56978 [M+H]^+^ on LTQ-FT Ultra MS using SIM mode at R = 100,000. Spectral accuracy was calculated at 90.3 using MassWorks^TM^. With the combinatory help of MassWorks^TM^ and online databases such as HMD, the component was putatively identified as phosphotidylcholine PC34:2. For validation purpose, by the help of Mass Frontier^TM^, the fragmentation pattern was elucidated explaining the MS/MS mass spectra very well (Figure 3). For further validation, a commercially available synthetic standard was analyzed on HILIC LC-MS (Figure 4). Four isomers were found in this standard, suggesting that the standard was not pure. However, biological roles of these isomers, as well as stereochemistry, require further investigation. 

**Table 1:** Putative biomarkers for pancreatic ductal adenocarcinoma (PDAC).

Substance Name	PubChem CID	m/z-Ion polarity	Profiling Method	p-Value	Fold Change
*Increased in PDAC*					
Arachidonic acid	444899		GC-TOF-MS	0.040982	1.49
Erythritol	8998		GC-TOF-MS	0.008525	1.53
Cholesterol	5997		GC-TOF-MS	0.030047	1.85
N-Methylalanine	5288725		GC-TOF-MS	0.024311	2.81
Lysine	5962	147.1 pos	HILIC-LC/MS	0.017356	1.03
Deoxycholylglycine	9675	448.53 neg	HILIC/RP-LC/MS	0.000052	1.31
Cholylglycine	16219399	464.42 neg	HILIC/RP-LC/MS	0.000001	2.61
LysoPC (16:0)	86554	496.2 pos	HILIC-LC/MS	0.000645	1.33
Tauroursodeoxycholic	3034759	498.34 neg	RP-LC/MS	0.004029	2.01
Taurocholic acid	6675	514.1 neg	HILIC/RP-LC/MS	0.000312	1.75
LysoPC(18:2)	11988421	520.23 pos	RP-LC/MS	0.013425	1.59
PE(26:0)	9546763	606.23 neg	RP-LC/MS	0.012072	1.81
PC (34:2)	6021688	758.31 pos	HILIC-LC/MS	0.008014	1.32
Unknown		753.12 pos	HILIC-LC/MS	0.000002	1.27
Unknown		265.07 pos	HILIC-LC/MS	0.000005	1.17
Unknown		332.07 pos	HILIC-LC/MS	0.000031	1.37
Unknown		633.19 pos	RP-LC/MS	0.009372	1.8
Unknown		414.15 pos	RP-LC/MS	0.006497	1.69
*Decreased in PDAC*					
Glutamine	5961	145.22 neg	HILIC/RP-LC/MS	0.000021	1.2
Hydrocinnamic acid	107	149.12 neg	HILIC-LC/MS	0.000252	1.38
Phenylalanine	6140	166.12 pos	RP-LC/MS	0.036583	1.15
Tryptamine	1150	205.09 pos	RP-LC/MS	0.016353	1.07
Inosine	6021	267.21 neg	RP-LC/MS	0.000014	1.4
Unknown		187.12 neg	RP-LC/MS	0.000246	1.11

#### 2.3.4. Metabolite Network Analysis

**Figure 5:**
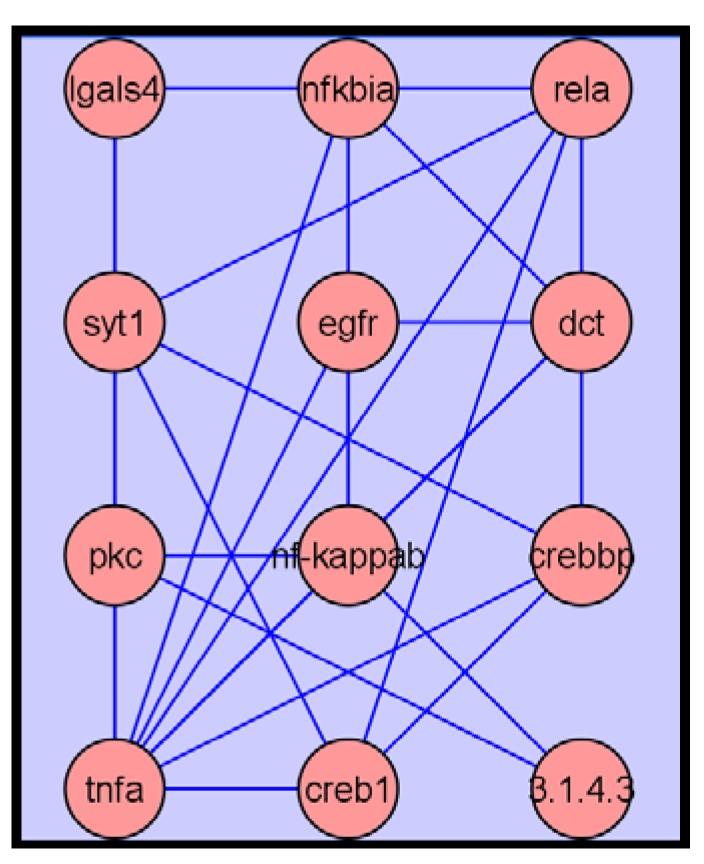
Network interactions depicted among putative biomarkers showing that tumor necrosis factor-α (TNF-α) and nuclear factor κB (NF-κB) genes had the most connections to other genes.

The putative PDAC biomarker list currently created was used for metabolite network analysis, providing valuable information about gene-protein-metabolite interactions and potential clinical correlates. The metabolite network graph illustrated that many genes were interwoven heavily and centered on tumor necrosis factor-α (TNF-α) and nuclear factor κB (NF-κB) genes, suggesting that products of these genes and related signaling pathways were significantly affected in PDAC patients (Figure 5).

Group 1—PDAC patients, Group 2—controls

#### 2.3.5. Validation

Identification of unknown metabolites is a challenging task. In the current study we annotated metabolites by collecting as much relevant structural information as possible. Due to limited plasma sample size, it is difficult to isolate unknown potential biomarkers for NMR experiments. Future studies should be designed to collect large amount of plasma, separate and purify plasma using preparative LC columns, and validate the structure of proposed putative biomarkers by accurate mass ion tree experiment using direct infusion nano-ESI FT ICR MS and by NMR experiments.

Because PDAC is uncommon as an inflammatory disease compared to other pro-inflammatory states such as cardiovascular diseases and metabolic syndrome, it is essential to select appropriate controls and validate proposed putative biomarkers by MRM experiments using LC-MS/MS in independent cohort studies controlled with other pro-inflammatory states like cardiovascular diseases and metabolic syndrome to eliminate confounding factors induced by these inflammatory conditions. Nevertheless, there is an on-going prospective study involving more than 100 PDAC patients and 100 controls. Nineteen identified compounds from the feature list of the current pilot study were semi-quantified using MRM on LC-MS/MS. Untargeted profiling was applied using GC-MS [81,82]. Results will be published separately in the future.

#### 2.3.6. Summary

Using comprehensive mass spectrometry based untargeted metabolomics we successfully found potential classifiers for pancreatic cancer in human plasma. A mechanism hypothesis was proposed based on pilot unbiased network analysis and follow-up validation studies. After validation with further cohort studies, some valid classifiers could become clinical biomarkers for early diagnosis of pancreatic cancer. We also found that HILIC LC-ESI-MS was useful at detecting naturally occurring isomers of plasma polar lipids.

## 3. The Application in Early Diagnosis of Kidney Cancer

### 3.1. Introduction

The most common type of kidney malignancy is clear cell renal cell carcinoma (ccRCC), which is frequently associated with mutations of the von Hippel-Lindau gene. ccRCC accounts for approximately 3% of adult malignancies and 90-95% of neoplasms arising from the kidney. In addition, ccRCC lacks early warning signs and is resistant to radiation and chemotherapy, demonstrating the need for early ccRCC diagnosis. Urine is ideally suited for metabolomic analysis, especially involving diseases of the kidney and urinary system [22,83]. In this case study with previously published data, the workflow of untargeted metabolomics is described to discover novel biomarkers of kidney cancer [25].

### 3.2. Procedure

#### 3.2.1. Sample Preparation

After urine was thawed on ice, neat urine sample was mixed with equal volumes of ice-cooled acetonitrile/methanol (v/v, 1:1) (methanol was added to extract more polar compounds). After sitting on ice for at least 5 min, sample was centrifuged for 5 min at 1,000 g, and supernatant was transferred to another tube. Methanol and acetonitrile were removed by Speed-Vac, and the aqueous leftover was dried with lyophilization process. For GC-MS, derivatization without further pretreatment was done even though urea peak may overload the capillary GC column, because severe artifacts occurred when using urease treatments. For LC-MS, sample was reconstituted in 100 µL water-acetonitrile (1:1, v/v) and the clear supernatant was transferred to a HPLC vial.

#### 3.2.2. Untargeted Screening Using Full Scan Mode (See Section 2)

#### 3.2.3. Subclass Screening Using Neutral Loss Scan, Precursor Ion Scan, and Predictive MRM

HPLC method was the same as described earlier. The entire effluent from the LC column was directed into the ESI source of an API 4000 Qtrap hybrid triple quadruple linear ion trap mass spectrometer (Applied Biosystems/MDS Sciex, Foster City, CA, USA) equipped with a TurboIonSpray source (heated electrospray source with an orthogonal source of heated gas to help desolvate the spray). Ion source parameters were set manually. The scales of the horizontal and vertical axes of the ion source were set at 6. Final TurboIonSource parameters were: curtain gas (CUR), 20 psi; collision gas (CAD), high; ionSpray voltage (IS), 5.2 kV; temperature (TEM), 300 °C; ion source gas 1 (GS1), 50 psi; ion source gas 2 (GS2), 50 psi; and interface heater (IHE), turned on.

Using parent compound of interest as the reference for tuning, compound-specific parameters were automatically optimized in LightSight^TM^ (version 2.0, Applied Biosystems/MDS Sciex). Using clomazone as an example, the automatic method creation tool in LightSight^TM^ was used to generate the following: (1) positive mode: Predictive Multiple Reaction Monitoring (pMRM-IDA), EMS full scan, precursor scan of *m/z* 125 (the fingerprint fragment of clomazone), precursor scan of *m/z* 141 (hydroxyl group added on the fingerprint fragment of clomazone), neutral loss scan of *m/z* 115 (the molecular ion of clomazone is *m/z* 240, minus the fingerprint fragment of *m/z* 125), neutral loss scan of *m/z* 129 for unknown glutathione conjugates; (2) negative mode: neutral loss scan of *m/z* 176 for unknown O-glucuronides, precursor scan of *m/z* 272 for unknown glutathione conjugates. Among these methods, the pMRM-IDA method was the most sensitive and typically used.

Parameters for the MRM scan were: declustering potential (DP): 61 V; entrance potential (EP): 10 V; collision energy (CE): 29 V; collision cell exit potential (CXP): 20 V. Q1 was set as unit resolution and Q3 as low resolution. Dwell time of each MRM channel was 5 msec and pause time was 2.5 msec. IDA criteria were set as the most intense ion exceeding 500 counts triggering an EPI scan to confirm charge state and/or isotope pattern selection. Parameters for EPI were: scan mode: profile, scan rate: 4,000 amu/s, LIT fill time: 5 msec, dynamic fill time: on, declustering potential (DP): 40 V; CES: 25 V; collision energy (CE): 60 V; collision cell exit potential (CXP): 20 V. Q1 was set as unit resolution.

### 3.3. Results and Discussion

#### 3.3.1. Untargeted Profiling

**Figure 6:**
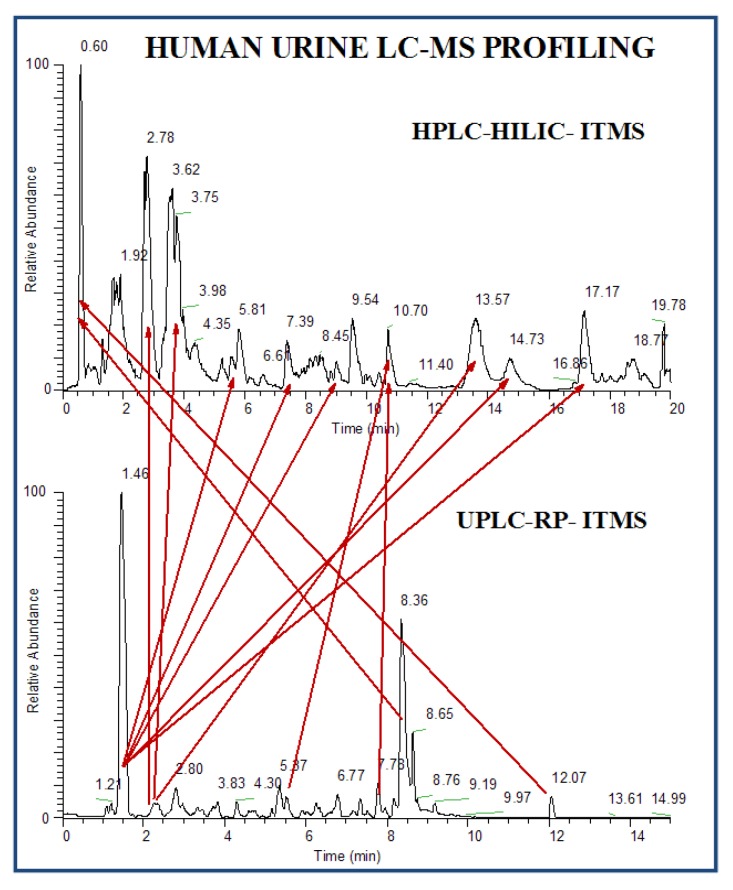
Comparison of LC-MS chromatograms of human urine sample acquired in RP and HILIC liquid chromatography modes on LTQ (linear ion trap mass spectrometer).

**Figure 7:**
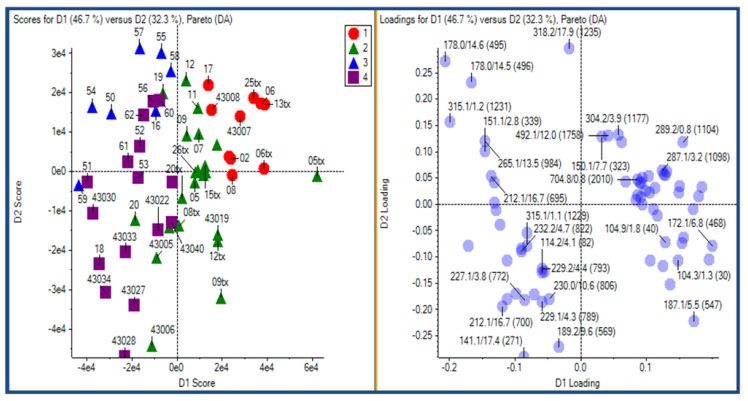
PCA-DA plots of the clear cell renal cell carcinoma (ccRCC) HILICLC–ESI–MS after manual feature selection.

**Figure 8:**
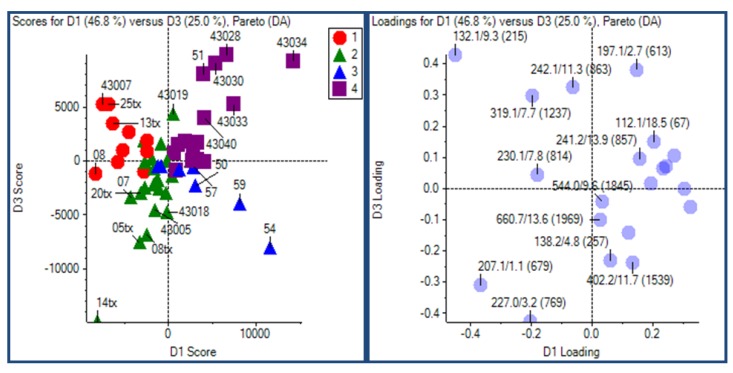
PCA-DA plots of the ccRCC HILIC-LC–ESI–MS after genetic algorithms (GA) feature selection.

**Figure 9:**
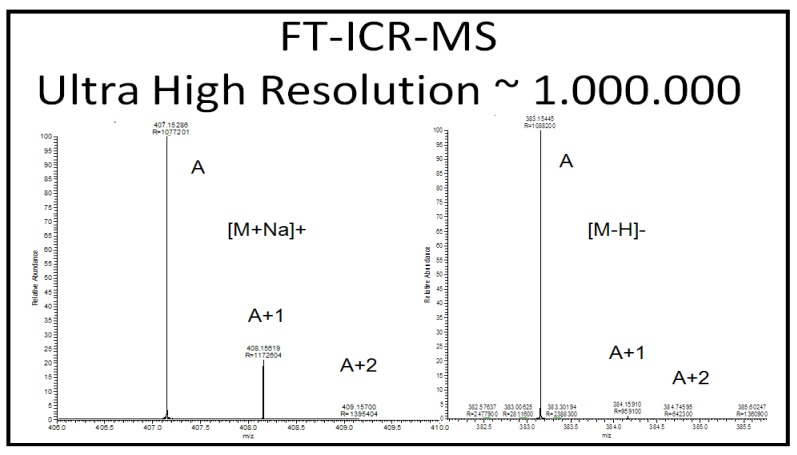
High-resolution spectra of a potential predictor, *m/z* 407.1527 at positive mode [M+Na]^+^ and *m/z* 383.1545 at negative mode [M-H]^−^, illustrating isotopic pattern at 1,000,000 resolution using NanoMate nano ESI-LTQ FT Ultra MS.

**Figure 10:**
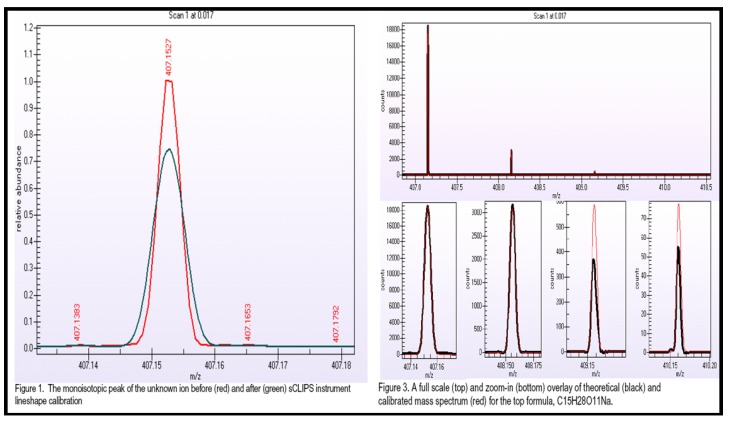
The unique elemental composition of m/z 407.1527 was assigned as C_15_H_28_O_11_Na using Mass Works^TM^ based on LC-MS data acquired using HPLC-LTQ FT Ultra MS at 100,000 resolution.

HILIC-LC-ESI-MS technique was more informative than the other two technology platforms in analyzing human urine samples. Therefore, only HILIC LC-MS dataset was presented. Most compounds in human urine are polar compounds, so that they may be more suitable for HILIC- than for RP-LC separations (Figure 6). Both GA and manual feature selection approaches were applied to peak tables generated with MarkerView^TM^. Different feature panels were found from these two independent feature selection approaches, whereas both were able to differentiate nicely ccRCC patients from healthy controls (Figure 7, Figure 8). Majority of GA selected predictors were relatively low abundant peaks, different from manually selected high abundant predictors. This observation is somewhat in accordance with our previous results and those described for NMR based metabolomics study where GA was applied for feature selection [84]. As an example, one putative biomarker was identified utilizing high-resolution FT-ICR MS for accurate mass measurement, Mass Works^TM^ for spectral accuracy and isotopic abundance pattern, and Mass Frontier^TM^ for fragmentation pattern (not shown) (Figure 9, Figure 10).

Groups were color-coded as: 1—red: female ccRCC, 2—green: male ccRCC, 3—blue: female control, 4—purple: male control. 55 samples, 68 predictors.

Groups were color-coded as: 1—red: female ccRCC, 2—green: male ccRCC, 3—blue: female control, 4-purple: male control. 55 samples, 22 predictors.

#### 3.3.2. Low Abundant Subclass Screening

**Figure 11:**
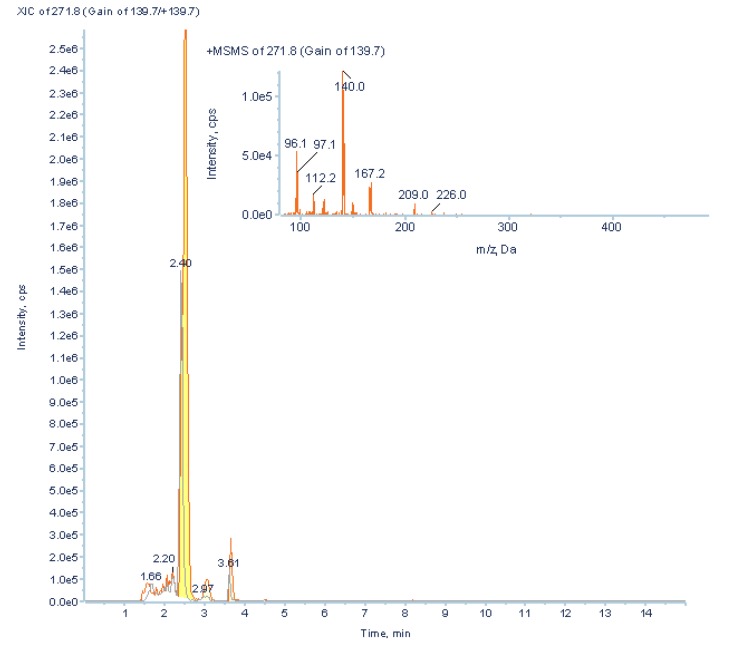
A nucleoside, putatively identified as N-formyl-cytidine (*m/z* 272, RT 2.52 min), was found using positive neutral loss scan of *m/z* 132 on Acquity UPLC-4000 QTrap MS in pooled urine from ccRCC patients (**orange line**) two-folds higher than in post-operative controls (**grey line**)

**Figure 12:**
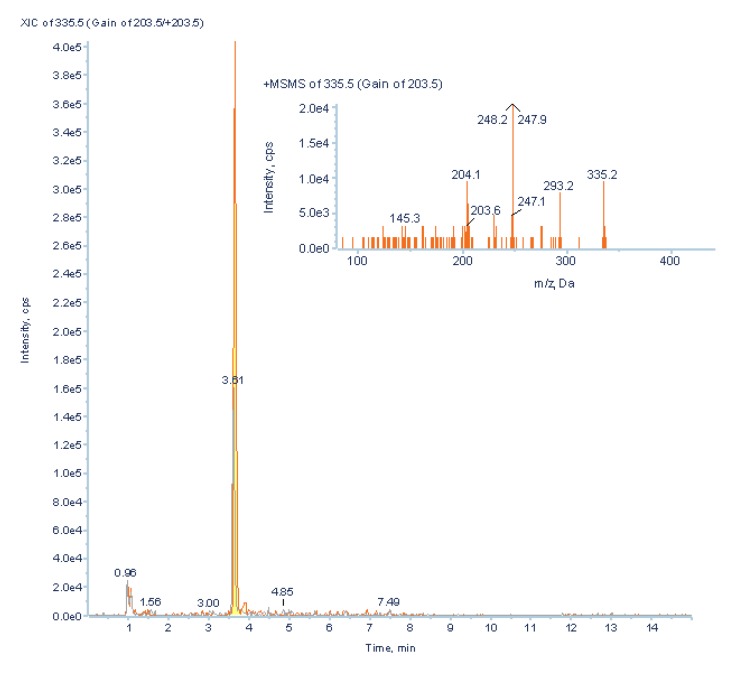
A nucleoside, putatively identified as N-butenoyl-guanosine (*m/z* 336, RT 3.65 min), was found using positive neutral loss scan of *m/z* 132 on Acquity UPLC-4000 QTrap MS in pooled urine from ccRCC patients (**orange line**) two-folds higher than in post-operative controls (**grey line**).

Although PCA differentiated between ccRCC patients and healthy controls, putative biomarkers were mostly involved in energy metabolism, suggesting low specificity of these potential biomarkers. In order to find more cancer-specific low abundant potential biomarkers, LightSight^TM^ was used to generate a positive mode method with neutral loss scan of 132 (loss of the ribose moiety) to screen for unknown RNA adducts. The method was composed of one neutral loss (NL) scan, one IDA criterion, and one enhanced product ion (EPI) scan [43]. Both pooled neat urine from cancer patients and post-operative controls were directly injected in Acquity UPLC-4000 QTrap MS/MS system. Several low abundant nucleoside metabolites were found and putatively identified (Figure 11, Figure 12, unpublished data).

#### 3.3.3. Summary

HILIC LC-MS profiling provided the most prominent clustering and biomarker discovery among the three technology platforms. Based on PCA visualization, ccRCC patients were quite different from healthy controls. Neutral loss scan was able to find low abundant RNA adducts that might be more specific to ccRCC.

## 4. Conclusions

When analyzing human blood plasma or urine for metabolomics studies, HILIC LC-ESI-MS is better than RP LC-ESI-MS and GC-MS in differentiation of cancer patients and healthy control subjects, whereas the latter two also provide unique feature components respectively. Parameter optimization for data pre-processing and data mining is extremely important. Different feature selection methods generate different panels of predictors and are good at discrimination of cancer and control groups, therefore final list should be the combination of the results of all feature selection approaches. Utilizing FT-ICR MS, accurate mass, isotope pattern, MS/MS fragmentation, and accurate mass MS^n^ ion tree experiments are extremely powerful at excluding confounding candidates and finally getting the unique elemental formula of unknown feature components. After identification of unknown feature components, metabolic network analysis can be done and is shown to be a very powerful tool to elucidate underlying biological mechanisms. Low abundant metabolites are as important or discriminative as high abundant metabolites. Discovery of low abundant differentiators requires different types of instrumentation and methodology such as predictive MRM, neutral loss scan, precursor ion scan, *etc.* Currently, fast *de novo* identification of multiple unknown feature components and untargeted profiling of trace-level differentiators are two of the most challenging issues in the field of metabolomics. General conclusion is that instrumental analysis and data mining should not be limited to single platform or algorithm since important information may be overlooked.

The above described comprehensive metabolomics workflow has been applied in blood plasma of pancreatic cancer patients and urine of kidney cancer patients in small scale case-control studies. In order to translate the results of these preliminary metabolomics studies into clinical applications, different levels of validation are required. First, the putative biomarkers obtained from small scale preliminary experiments should be validated with metabolomics studies of much larger sample size and designed to exclude confounding factors. For large-scale metabolomics studies, quality control and assurance is essential because the samples cannot be analyzed in one batch and in one day, therefore, errors from instrumental drifting and sample preparation need to be monitored and corrected. Second, those validated feature components are *de novo* identified so that MRM quantitation methods can be established. MRM quantitation is used for validation of the identified biomarkers in large-scale prospective epidemiological studies or double-blinded clinical trials. Finally, if these potential biomarkers are valid as surrogates of disease status, causal relationship in cancer pathophysiology is then investigated in preclinical mechanism studies using *in vitro* cell-based models and *in vivo* animal models.
